# SALL4 induces radioresistance in nasopharyngeal carcinoma via the ATM/Chk2/p53 pathway

**DOI:** 10.1002/cam4.2056

**Published:** 2019-03-24

**Authors:** Xin Nie, Ergang Guo, Cheng Wu, Dongbo Liu, Wei Sun, Linli Zhang, Guoxian Long, Qi Mei, Kongming Wu, Huihua Xiong, Guoqing Hu

**Affiliations:** ^1^ Department of Oncology Tongji Hospital Tongji Medical College Huazhong University of Science and Technology Wuhan Hubei Province China

**Keywords:** apoptosis, cell cycle, nasopharyngeal carcinoma, radioresistance, SALL4

## Abstract

Radiotherapy is the mainstay and primary curative treatment modality in nasopharyngeal carcinoma (NPC), whose efficacy is limited by the development of intrinsic and acquired radioresistance. Thus, deciphering new molecular targets and pathways is essential for enhancing the radiosensitivity of NPC. SALL4 is a vital factor in the development and prognosis of various cancers, but its role in radioresistance remains elusive. This study aimed to explore the association of SALL4 expression with radioresistance of NPC. It was revealed that SALL4 expression was closely correlated with advanced T classification of NPC patients. Inhibition of SALL4 reduced proliferation and sensitized cells to radiation both in vitro and in vivo. Furthermore, SALL4 silencing increased radiation‐induced DNA damage, apoptosis, and G2/M arrest in CNE2 and CNE2R cells. Moreover, knockdown of SALL4 impaired the expression of p‐ATM, p‐Chk2, p‐p53, and anti‐apoptosis protein Bcl‐2, while pro‐apoptosis protein was upregulated. These findings indicate that SALL4 could induce radioresistance via ATM/Chk2/p53 pathway and its downstream proteins related to apoptosis. Targeting SALL4 might be a promising approach for the development of novel radiosensitizing therapeutic agents for radioresistant NPC patients.

## 1 INTRODUCTION

Nasopharyngeal carcinoma (NPC) is the most common malignancy originating in the nasopharynx cavity, which has profound geographical and is a unique disease endemic in Southeast Asia.^1^ In 2014, the age‐standardized incidence rates (per 100 000 population) to Chinese standard population and to world standard population were 2.48 and 2.33, respectively.^2^ As a staple cancer treatment approach, radiotherapy (RT) is the mainstay and primary curative treatment modality in NPC. However, the RT efficacy is limited by the development of radiation resistance, which is still the major obstacle to achieve long‐term survivals.^3^ Therefore, deciphering new molecular targets and pathways that mediate radioresistance is crucial and urgent to enhance RT efficacy in NPC.

As a member of the mammal homologs of *Drosophila* homeotic gene *spalt* (sal), SALL4 (sal‐like 4) is ubiquitously expressed in the embryo, but rarely expressed in adult cells after birth.^4^ However, a large body of studies has demonstrated restored SALL4 expression in malignancies.^5^, ^6^, ^7^, ^8^, ^9^, ^10^, ^11^, ^12^ Abnormal expression of SALL4 promotes tumorigenesis, invasion and metastasis through maintaining cancer stem cell properties.^13^ What's more, a recent study indicated that the stemness factor SALL4 was required for efficient recruitment and activation of Ataxia‐telangiectasia mutated kinase (ATM), which is the initiator of DNA damage response.^14^ Meanwhile, the inhibition of SALL4 could induce apoptosis and cell cycle arrest in several cancers.^15^, ^16^ Although DNA damage and cellular apoptosis were the main therapeutic effects of radiation on cancer cells,^17^, ^18^ the correlation between SALL4 and radiosensitivity remains unclear, and the underlying mechanisms need to be explored.

In the DNA damage responses (DDRs), a complex network of proteins is required for cell cycle checkpoint and DNA repair.^19^ ATM/Chk2/p53 pathway is known to induce cell cycle arrest and activate the p53‐related apoptotic pathway in DDRs.^20^ ATM, a DNA damage initiator, is activated through autophosphorylation of the Ser1981, and then activates the distal transducer kinase checkpoint kinase 2 (Chk2), resulting in cell cycle checkpoint initiation and/or apoptosis.^19^, ^21^ The activated cell cycle checkpoints provide more time for DNA repair.^17^ But if related proteins were dysfunctional, balance is disrupted between cell proliferation and cell death, and ultimately results in cellular growth arrest or death.^22^, ^23^ Loss of ATM or Chk2 expression or inhibition of their kinase activity could sensitize cells to death.^24^ A study in gynecologic cancer cells also found that inhibition of ATM enhanced the cellular response to radiation.^25^ In addition, Xiong et al. confirmed that, following irradiation, impaired autophosphorylation of ATM in Sall4^−/−^ embryonic stem cells (ESCs) are caused by the loss of SALL4.^14^ Taken together, we hypothesized that SALL4 might regulate radiosensitivity via ATM/Chk2/p53 pathway in NPC.

Therefore, the present study aimed to estimate the association of SALL4 expression in NPC samples and clinical stages. Moreover, in vivo and in vitro experiments were conducted to interfere SALL4 expression in order to evaluate its influence on radiosensitivity as well as the potential mechanism.

## 2 MATERIALS AND METHODS

### 2.1 Clinical samples

Tissue samples from 131 NPC patients were collected at Tongji Hospital of Tongji Medical College, Huazhong University of Science and Technology between Jan 2015 and Aug 2018. Additionally, 10 noncancerous nasopharyngeal samples from nasal polyps patients were also collected. The diagnosis of NPC for each patient was confirmed by two independent histopathologists. None of the NPC patients received radiotherapy, chemotherapy, or other medical interventions prior to biopsy. Then all NPC patients were classified according to the eighth edition of the American Joint Committee on Cancer (AJCC) Cancer Staging Manual.^26^ This study was approved by the Research Ethics Committee of the Tongji Hospital of Tongji Medical College, Huazhong University of Science and Technology.

### 2.2 Immunohistochemistry

The tissue specimens embedded in paraffin were sliced into 5 μm‐thick sections and all slides were stained for SALL4 (sc101147, 1:50 dilution; Santa Cruz Biotechnology) following the standard protocols. Then SALL4 expression was scored according to the staining scope and intensity. Specifically, staining scope: 1 (0%‐25%); 2 (25%‐50%); 3 (50%‐75%); and 4 (75%‐100%), and staining intensity: 0 (negative); 1 (weakly positive; light yellow); 2 (moderately positive; yellow‐brown), and 3 (strongly positive; dark brown). SALL4 expression = staining scope × intensity. Then, expression score ≥6 was defined as high expression, and those <6 was defined as low expression.^27^

### 2.3 Cell culture

Human NPC cell lines (CNE1, CNE2, and CNE1‐LMP1) were a gift from the Cancer Research Institute of Central South University (Changsha, China). The CNE2‐radioresistance (CNE2R) cell line was constructed from a poorly differentiated CNE2 cell line by exposing to progressively increasing radiation over the course of 6 months. Briefly, CNE2 cells were irradiated every 2 weeks with 2, 4, 6, and 8 Gy, and each dose was repeated for three times so that the CNE2 cells were exposed to a total dose of 60 Gy. CNE2 and CNE2R cell lines have been authenticated using STR DNA profiling analysis. All NPC cell lines were cultured in RPMI‐1640 medium (KeyGEN, Jiangsu, China) containing 10% fetal bovine serum (FBS, Gibco Life Technologies, Grand Island, NY, USA) and 1% penicillin‐streptomycin solution (Hyclone, Thermo Scientific, Marietta, OH, USA) at 37°C in 5% CO_2_. Human breast carcinoma cell line MCF7 and lung cancer cell line A549 were maintained in our laboratory and grown in DMEM medium (KeyGEN, Jiangsu, China) containing 10% FBS and 1% penicillin‐streptomycin solution at 37°C in 5% CO_2_.

### 2.4 Colony formation assay

Cells were seeded into 6‐well plates and irradiated with indicated doses (0, 2, 4, 6, 8, and 10 Gy). Approximately 10‐14 days later, colonies were fixed with 100% methanol (SCR, Shanghai, China) and stained with 0.1% crystal violet (SCR, Shanghai, China) for 15 minutes. The plates were photographed and colonies consisting of 50 or more cells were counted. Then plating efficiency (PE) was calculated using the following formula: PE = number of colonies/number of cells seeded (0 Gy), and survival fraction (SF) of each group was determined using the equation: SF = colony number/(plating cell number × PE). The SF curve was calculated according to the multi‐target single‐hit model SF = 1 − (1−e^−D/D0^)^N^, and the radiobiological parameters SF_2_ (SF of 2 Gy), D_0_ (mean lethal dose or final slope), D_q_ (quasi‐threshold dose), and N (extrapolation number) were determined using this model. Additionally, the radiation sensitivity enhancement ratio (SER) was calculated using the formula: SER = D_0_ of the control group/D_0_ of the transfection group.^28^ Three parallel samples were set at each radiation dosage.

### 2.5 Lentiviral transfection

The sh‐SALL4 lentiviral particle, SALL4‐plasmid lentiviral particle, and the empty‐vector lentiviral particles were purchased from GeneChem (Shanghai, China). Lentiviruses were transfected into CNE2 and CNE2R cell lines according to the manufacturer's protocol. The stably transfected cells were selected by adding 2 μg/ml puromycin (Sigma, St Louis, USA) into the medium for at least 2 weeks. Western blot was applied to analyze the expression of SALL4.

### 2.6 RNA extraction and quantitative real‐time PCR

Total RNA was extracted using RNAiso Plus (Takara, Dalian, China) and reverse‐transcribed into cDNA using the PrimeScript RT Reagent kit (RR037A; Takara, Dalian, China) according to the manufacturer's introduction. Then quantitative real‐time PCR (qRT‐PCR) was carried out using SYBR Premix Ex Taq (RR420A; Takara, Dalian, China) and a StepOne^™^ Real‐Time PCR system (ABI, CA, USA), and the final concentration of all reagents in the reaction and cycling conditions were set according to the manufacturer's introduction. The primer sequences were: GAPDH (forward: TGT ACG CCA ACA CAG TGC TG; reverse: TCA GGA GGA GCA ATG ATC TTG) and SALL4 (forward: AGT ATC AGA GCC GAA GCC CAG A; reverse: GGG CTC GGA TAA ACG TGG AA). Lastly, the ΔΔCq calculation method was used for the relative quantification of gene expression.^29^

### 2.7 Western blot assay

Cells were washed three times with ice‐cold PBS and lysed by RIPA lysis buffer (Beyotime, Shanghai, China) for 30 min on ice. Then, the whole‐cell lysates were centrifuged at 4°C, 12 000 *g* for 20 min and the protein was collected. The protein concentration was measured by the BCA protein assay kit (Beyotime, Shanghai, China). Equal amounts of denatured proteins were separated by 10% SDS‐PAGE gels and transferred to PVDF membranes (Millipore, Billerica, USA). The membranes were blocked with 5% skim milk or BSA for 1 h at room temperature, incubated with primary antibodies including ATM rabbit monoclonal antibody (RabMAb, #2873), p‐ATM (Ser1981) RabMAb (#5883), Chk2 rabbit polyclonal antibody (RabPAb, #2662), p‐Chk2 (Thr68) RabPAb (#2661), p53 RabMAb (#2527), p‐p53 (Ser15) RabPAb (#9284), cleaved caspase‐3 RabMAb (#9664) (1:1000 dilution, Cell Signaling Technology, Beverly, MA, USA), Bax RabPAb (#50599‐2‐Ig), Bcl‐2 RabPAb (#12789‐1‐AP) (1:1000 dilution, Proteintech, Wuhan, China), GAPDH mouse monoclonal antibody (MoMAb, #60004‐1‐Ig), β‐actin MoMAb (#60008‐1‐Ig) (1:5000 dilution, Proteintech, Wuhan, China), and SALL4 MoMAb (#sc101147, 1:200 dilution, Santa Cruz Biotechnology, CA, USA) at 4°C overnight, and then incubated with corresponding secondary antibodies (1:5000 dilution, Boster, Wuhan, China) for 1 hour at room temperature. Finally, the immunoblots were detected by ECL kit (Thermo Scientific, Marietta, OH, USA). Images were captured with SynGene G: Box Chemi XRQ (Alpha Metrix Biotech, Rödermark, Hesse, Germany), and intensity of blot bands was analyzed by ImageJ 1.8.0 (National Institutes of Health, Bethesda, MA, USA).

### 2.8 Cell counting kit‐8 (CCK8) assay

Cell counting kit‐8 assay (Promoter, Wuhan, China) was used for assessing the cell proliferation ability. Briefly, cells were plated at a concentration of 800 cells/well in 96‐well plates, and then cell viability was assessed daily for 6 days by adding 10 μL CCK8 solutions to each well and incubated for another 2 hours at 37°C. The absorbance at 450 nm was measured by the microplate reader (BioTek, Winooski, VT, USA).

### 2.9 Immunofluorescence assay

Cells were placed on sterile coverslips in 24‐well plates at a concentration of 2 × 10^4^ cells/well and irradiated with 6 Gy. At 30 minutes after radiation, coverslips were fixed in 4% paraformaldehyde, permeabilized with 0.1% Triton X‐100 (Servicebio, Wuhan, China), blocked with 0.5% BSA (Sigma, St. Louis, MO, USA), incubated with the γ‐H2AX RabMAb (#9718, 1:400 dilution, Cell Signaling Technology, Beverly, MA, USA), secondary antibody (#SA00006‐4, 1:500 dilution, Alexa Fluor 594, Proteintech, Wuhan, China), followed by conjugated stained with 4’, 6‐diamidino‐2‐phenylindole (DAPI, Boster, Wuhan, China). Following this, cells were visualized by laser scanning confocal microscopy (Zeiss, Germany). And images were analyzed using ImageJ 1.8.0.

### 2.10 Flow cytometry analysis

Flow cytometry was performed for apoptosis and cell cycle analysis. Cells were trypsinized and harvested at 72 hours post‐irradiation (10 Gy). The collected cells were stained with Annexin V‐FITC and PI following the manufacturer's protocol (BD Biosciences, Franklin Lakes, NJ, USA). For cell cycle distribution analysis, cells were fixed with 70% cold ethanol for 24 h and digested with RNase for 30 min at 37°C at 48 hours after irradiation of 6 Gy. Then cells were stained with propidium iodide (PI) for 30 min at 4°C according to the manufacturer's protocol (Promoter, Wuhan, China). The samples were subjected to flow cytometry using LSRFortessa (BD Biosciences, San Jose, CA), and data were analyzed using FlowJo version 10 (TreeStar, San Diego, CA, USA).

### 2.11 Animals

Five‐week‐old BALB/c nude mice were used for the construction of the xenograft model in vivo. Cells (1 × 10^7^/200 μL PBS) were inoculated subcutaneously into left flanks of each mouse, which were randomized into eight groups with six animals in each group: 1) the untreated CNE2‐CTL group; 2) the untreated CNE2‐SALL4 group; 3) the CNE2‐CTL irradiation group, 4) the CNE2‐SALL4 irradiation group; 5) the untreated CNE2R‐CTL group; 6) the untreated CNE2R‐shSALL4 group; 7) the CNE2R‐CTL irradiation group and 8) the CNE2R‐shSALL4 irradiation group. When the tumor volume reached 100‐150 mm^3^, the tumor areas of mice in irradiation groups were irradiated with 8 Gy. Tumor sizes were recorded every 2 or 3 days. The tumor weight inhibition rates (TWI %) were calculated according to the formula: TWI % =  (1‐tumor weight of irradiation group/tumor weight of unirradiation group) × 100%.^30^ Procedures involving animals and their care were conducted in conformity with the ARRIVE (Animal Research: Reporting of In Vivo Experiments) guidelines and the AVMA (American Veterinary Medical Association) euthanasia guidelines, and were approved by the Animal Ethics Committee of Tongji Hospital, Tongji Medical College, Huazhong University of Science and Technology.

### 2.12 Statistical analysis

All in vitro experiments were performed in triplicate. Data were shown as mean ± SD. SPSS version 17.0 software was used, and *t* tests or ANOVA tests were applied to test the statistical significance. The chi‐square test was used to analyze the relationship between SALL4 expression and clinic‐pathological features. A value of *P *<* *0.05 was considered significant.

## 3 RESULTS

### 3.1 Associations of SALL4 expression with clinical and pathological characteristics of NPC patients

**Figure 1:**
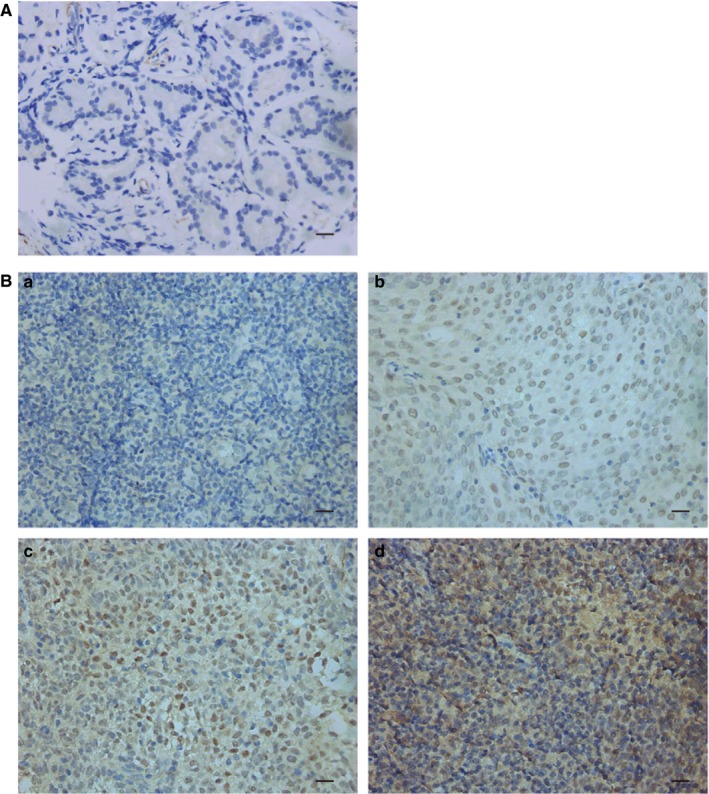
Representative immunohistochemistry staining image for SALL4 expression in NPC tissue (original magnification × 400; Bar, 20 μm). A, Representative image for negative expression in noncancerous tissues (nasal polyp). B, Representative images for different staining intensity of SALL4 in NPC tissues. a: negative; b: weakly positive (light yellow); c: moderately positive (yellow‐brown), and d: strongly positive (dark brown)

We observed that cancerous tissues displayed a relatively high level of SALL4 compared to noncancerous tissues (5.80 ± 0.284 vs 0 ± 0, *P *<* *0.001, Table 1, Figure 1). Generally, in cancerous tissues, the later cancer classification and stage were, the higher expression of SALL4 was. Patients in T3‐4 classification showed higher score of SALL4 expression, compared with those in T1‐2 classification (6.47 ± 0.347 vs 4.53 ± 0.441, *P *=* *0.001, Table 1). Moreover, patients in stage III‐IV had higher score of SALL4 expression than that in stage I‐II (6.10 ± 0.310 vs 4.05 ± 0.575, *P *=* *0.011, Table 1).

**Table 1:** The semiquantitative immunohistochemical score of SALL4 expression in noncancerous and cancerous tissues

Characteristics	Mean ± SEM^a^	No. of patients (%)	*P*‐value
Noncancerous tissues	0 ± 0	10 (100)	**0.000** ***
Cancerous tissues	5.80 ± 0.284	131 (100)	
Age			
<60	5.77 ± 0.330	104 (79.4)	0.824
>= 60	5.93 ± 0.544	27 (20.6)	
Gender			
Male	6.10 ± 0.360	87 (66.4)	0.136
Female	5.20 ± 0.450	44 (33.6)	
T classification			
T1‐2	4.53 ± 0.441	45 (34.3)	**0.001** **
T1	3.74 ± 0.729	19 (14.5)	
T2	5.12 ± 0.530	26 (19.8)	
T3‐4	6.47 ± 0.347	86 (65.7)	
T3	6.73 ± 0.510	44 (33.6)	
T4	6.19 ± 0.470	42 (32.1)	
N classification			
N0‐1	5.34 ± 0.473	50 (38.2)	0.203
N0	4.50 ± 0.861	16 (12.2)	
N1	5.74 ± 0.561	34 (26)	
N2‐3	6.09 ± 0.354	81 (61.8)	
N2	6.02 ± 0.431	58 (44.3)	
N3	6.26 ± 0.623	23 (17.6)	
M classification			
M0	5.71 ± 0.300	116 (88.5)	0.356
M1	6.53 ± 0.883	15 (11.5)	
Clinical stage			
I‐II	4.05 ± 0.575	19 (14.5)	**0.011** *
I	2.33 ± 1.856	3 (2.3)	
II	4.38 ± 0.584	16 (12.2)	
III‐IV	6.10 ± 0.310	112 (85.5)	
III	5.92 ± 0.509	49 (37.4)	
IV	6.24 ± 0.386	63 (48.1)	
Histologic subtypes			
WHO I	2.00 ± 1.000	2 (1.5)	>0.05
WHO II	5.78 ± 1.115	9 (6.9)	
WHO III	5.87 ± 0.296	120 (91.6)	

a standard error of the mean

***P <* *0.001, **P <* *0.01, *P <* *0.05.

To seek the clinical significance of SALL4 in NPC, immunohistochemistry was employed to assess the SALL4 expression in NPC samples, and chi‐square tests were performed to analyze the correlation between SALL4 expression and clinic‐pathological features of NPC patients. In detail, high expression of SALL4 was detected in 59.5% (78 of 131) of NPC patients (Table 2). SALL4 expression was not associated with patient age, gender, N classification, distant metastasis, clinical stage or histologic subtype, but correlated with T classification (*P *=* *0.030, Table 2). A higher proportion of high expression of SALL4 was showed in T3‐4 classification NPC patients (66.3% vs 46.7%, *P *=* *0.030, Table 2). Our findings demonstrated that SALL4 expression was remarkably correlated with the T classification.

**Table 2:** The association between SALL4 expression and clinic‐pathological characteristics of nasopharyngeal cancer patients

Characteristics	SALL4 expression		*P*‐value
	Low N (%)	High N (%)	
Age			
<60	43 (41.3)	61 (58.7)	0.684
>= 60	10 (37.0)	17 (63.0)	
Gender			
Male	33 (37.9)	54 (62.1)	0.407
Female	20 (45.5)	24 (54.5)	
T classification			
T1‐2	24 (53.3)	21 (46.7)	**0.030** d
T3‐4	29 (33.7)	57 (66.3)	
N classification			
N0‐1	25 (50.0)	25 (50.0)	0.080
N2‐3	28 (34.6)	53 (65.4)	
M classification			
M0	48 (41.4)	68 (58.6)	0.550
M1	5 (33.3)	10 (66.7)	
Clinical stage			
I‐II	11 (57.9)	8 (42.1)	0.094
III‐IV	42 (37.5)	70 (62.5)	
Histologic subtypes			
WHO type I^a^	2 (100)	0 (0)	0.214
WHO type II^b^	4 (44.4)	5 (55.6)	
WHO type III^c^	47 (39.2)	73 (60.8)	

a keratinizing squamous cell carcinoma

b nonkeratinizing differentiated carcinoma

c nonkeratinizing undifferentiated carcinoma

d *P *<* *0.05

### 3.2 Construction of acquired radioresistant cell line and interference of SALL4 expression in NPC cell lines

**Figure 2:**
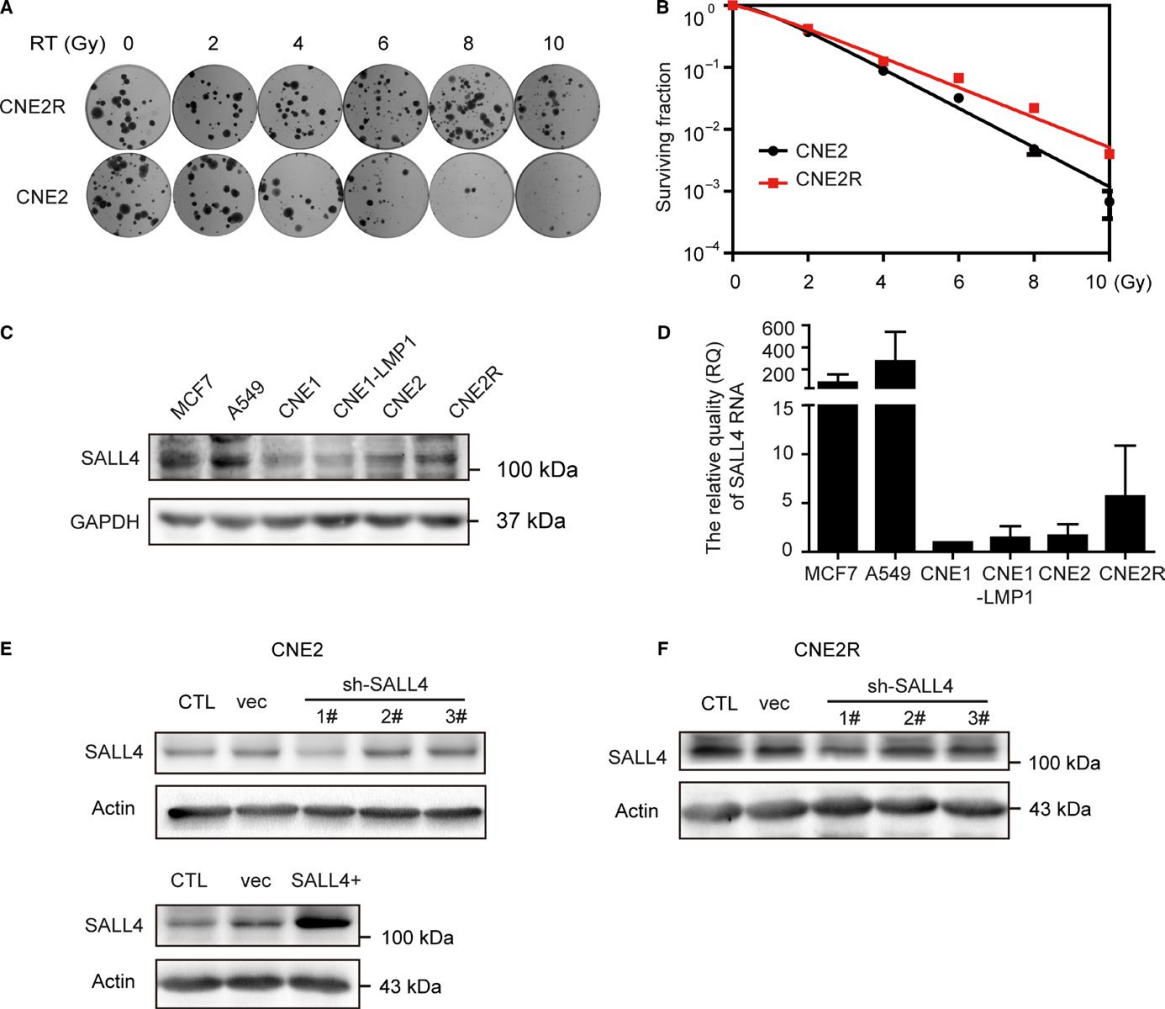
Construction of acquired radioresistant cell line CNE2R and interference of SALL4 expression in NPC cell lines. A, Representative photograph of colony formation assay. B, Survival fraction in CNE2R cells was markedly increased compared with those in CNE2 cells. C and D, Western blot assay and quantitative real‐time PCR were used to detect SALL4 expression in MCF7, A549 cells and NPC cell lines. GAPDH was used as a loading control. E and F, SALL4 expression was detected by western blot to analyze the effect of lentivirus transfection in CNE2 (E) and CNE2R (F) cells. β‐Actin was used as a loading control

First of all, the acquired radioresistant CNE2 cell line (CNE2R) was successfully generated from a poorly differentiated CNE2 cell line and its radioresistance was confirmed by colony formation assay. And the SER in CNE2R cells was 0.76‐fold change from the parental CNE2 cells (Table 3). Compared with parental cells, CNE2R cells had relatively higher colony survival after exposure to different doses of radiation (Figure 2A, B). Next, SALL4 expression was assessed by western blot and qRT‐PCR (Figure 2C, 2D). Because high expression of SALL4 has been reported in breast cancer cell MCF7 and lung cancer cell A549, these two cell lines were employed as positive controls.^31^, ^32^ Results showed that SALL4 expression of CNE2 cells was at a moderate level, whereas that of CNE2R cells was higher at both protein and RNA levels (Figure 2C, D). Then, for further exploring the functional role of SALL4 in radioresistance, we used lentivirus‐mediated systems to generate SALL4 silencing CNE2 and CNE2R cell lines (CNE2‐shSALL4 1# and CNE2R‐shSALL4 1#) and SALL4 stably expressing CNE2 cell line (CNE2‐SALL4) (Figure 2E, F). SALL4 expression of cells transfected with shSALL4 1# was inhibited mostly, and these cells were named as shSALL4 cells. Those cells were used for the following experiments.

**Table 3:** The radiobiological parameters of NPC cells exposed to radiation

Cell line	SF_2_	D_0_	D_q_	N	SER
CNE2	0.37	1.37	0.41	1.35	–
CNE2R	0.42	1.79	1.03	1.78	0.76
CNE2‐CTL	0.52	1.39	1.38	2.71	–
CNE2‐shSALL4	0.41	1.26	0.83	1.94	1.11
CNE2‐SALL4	0.60	1.81	1.49	2.29	0.77
CNE2R‐CTL	0.54	1.61	0.72	1.57	–
CNE2R‐shSALL4	0.42	1.41	0.62	1.55	1.14

### 3.3 SALL4 is correlated with cell proliferation, as well as the intrinsic and acquired radioresistance of NPC

**Figure 3:**
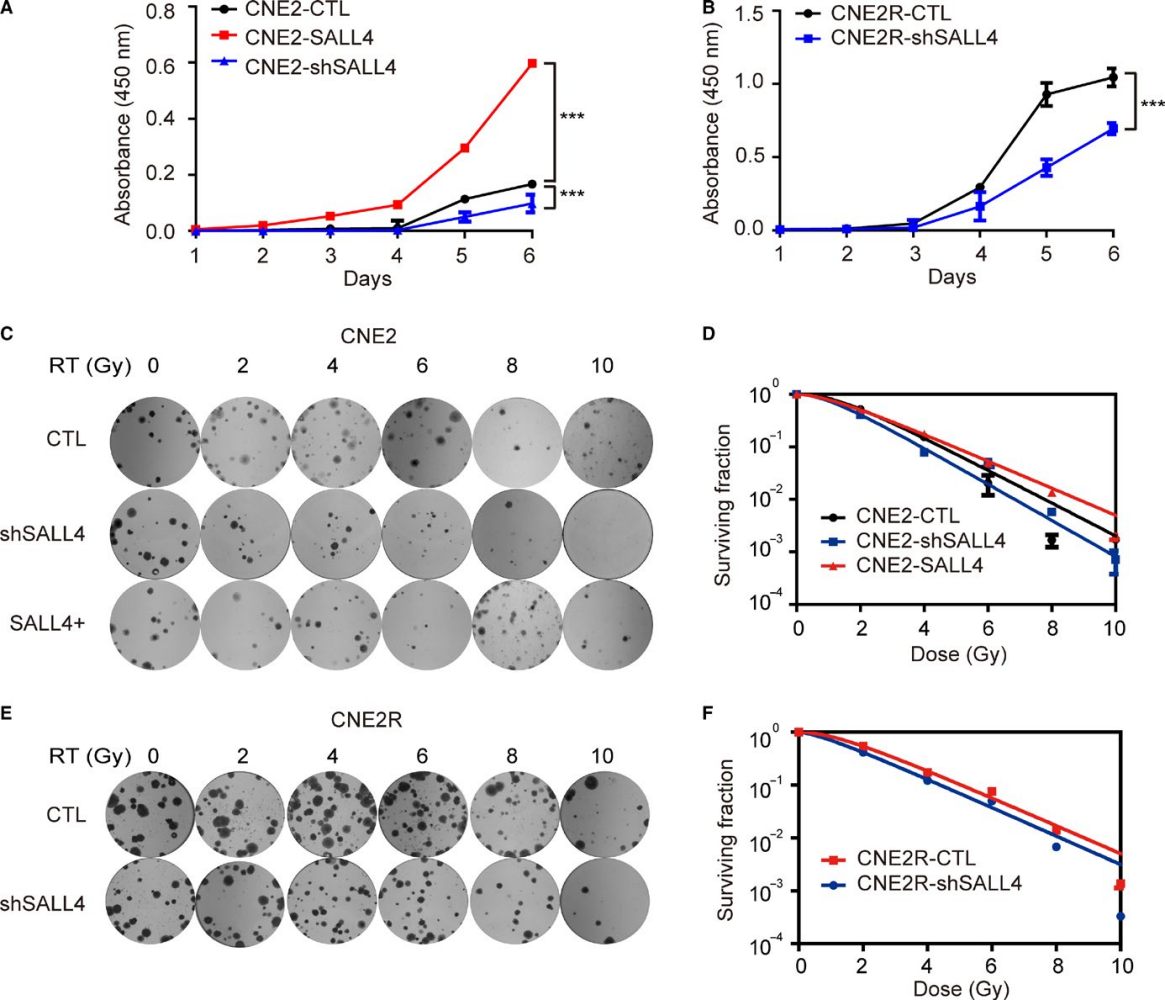
The effect of interference of SALL4 expression on proliferation and radiosensitivity. A and B, CCK8 assays were used to detect the proliferation ability. A, Silencing SALL4 reduced proliferation and SALL4 overexpression increased proliferation in CNE2 cells. B, Silencing SALL4 reduced the proliferation ability of CNE2R cells. Diagrams were from three independent experiments (mean ± SD, n = 3). ***P <* *0.001. C and E, Representative photographs of colony formation assays. D, Survival fractions of CNE2 cells were increased by overexpressing SALL4, and decreased by SALL4 silencing, compared to the control group. F, SALL4 silencing remarkably decreased the survival fraction of CNE2R cells, compared to control group

Cell counting kit‐8 assay and colony formation assays were used to investigate the association of SALL4 expression with proliferation and radiosensitivity of NPC cells. Results of CCK8 assay exhibited that cell proliferation was dramatically increased in CNE2‐SALL4 cells, but was forcefully decreased in CNE2‐shSALL4 cells and CNE2R‐shSALL4 cells, in contrast to control groups (Figure 3A, 3B). Here, the function of SALL4 in accelerating proliferation was confirmed again in vitro.

To further address the impact of SALL4 expression on cellular sensitivity to radiation, we performed colony formation assay and found that CNE2‐shSALL4 and CNE2R‐shSALL4 showed lower survival rate and decreased value of SF_2_ compared to control groups after irradiation (0.41 vs 0.52; 0.42 vs 0.54, Table 3; Figure 3C‐F). The SER in shSALL4 groups were 1.11‐fold and 1.14‐fold changes from the CNE2 and CNE2R control groups, respectively (Table 3), which suggested that SALL4 silencing had radiosensitization effect on the parental CNE2 cells and the radioresistant CNE2R cells. Consistently, CNE2‐SALL4 had higher survival rate and increased SF_2_ value compared to the control group (0.60 vs 0.52, Table 3; Figure 3C and 3D). The SER in SALL4 overexpression group was 0.77‐fold change from the CNE2 control group (Table 3), indicating that SALL4 had the effect of inducing radioresistance on CNE2 cells. These data indicated that SALL4 expression was correlated with radiosensitivity, and inhibition of SALL4 could reverse the intrinsic and acquired radioresistance of NPC.

### 3.4 SALL4 is associated with DNA damage repair, apoptosis, and cell cycle arrest in NPC cells

**Figure 4:**
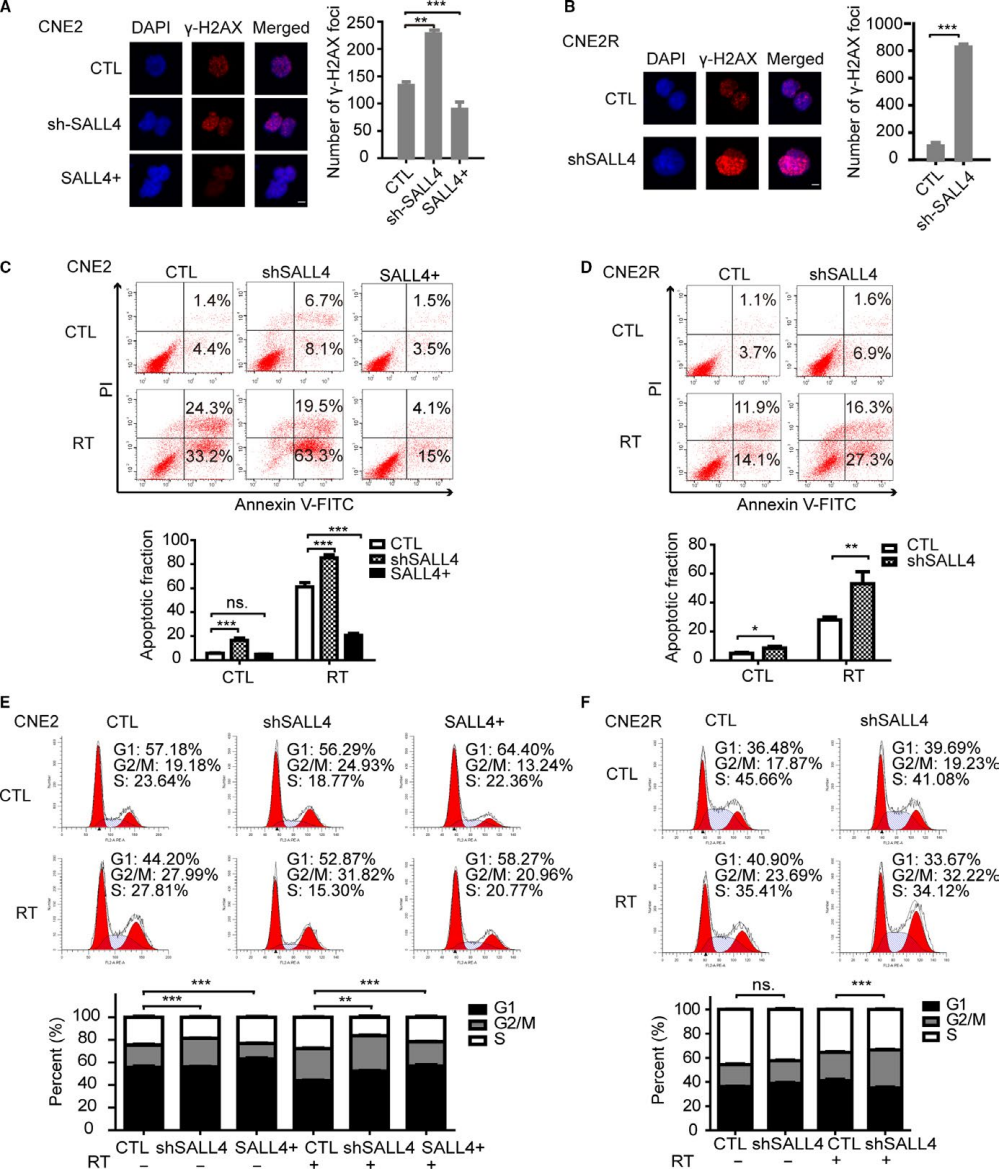
SALL4 is correlated with the γH2AX‐mediated repair of DNA double‐strand breaks (DSBs), apoptosis, and cell cycle arrest induced by radiation. A and B, Images were captured using confocal microscopy. Cell nucleus was stained with DAPI (blue) and antibody to γ‐H2AX (red). Bar, 5 μm. The number of γH2AX foci per cell was determined by analyzing 100 randomly selected cells. C and D, The apoptotic rates were determined by flow cytometry. Compared with control groups, the apoptotic rate decreased in SALL4 overexpression group, while that in SALL4 silencing groups increased. E and F, The cell cycle proportion was detected by flow cytometry. Knockdown of SALLL4 induced G2/M arrest, and SALL4 overexpression decreased G2/M proportion. All histograms were from three independent experiments (mean ± SD, n = 3). *P <* *0.05, **P <* *0.01 and ***P <* *0.001. ns: not significant

To search the mechanisms involved in SALL4‐induced radioresistance, cell immunofluorescence assay was used to detect the expression of phosphorylated histone H2AX (γ‐H2AX), the marker of DNA damage.^33^ After irradiation, the number of γ‐H2AX was higher in CNE2‐shSALL4, but lower in CNE2‐SALL4 cells than the control group (Figure 4A). Consistent with this finding, the number of γ‐H2AX significantly increased in CNE2R‐shSALL4 cells (*P *<* *0.001, Figure 4B). Those consequences were in support of the notion that SALL4 could impair radiation‐induced DNA damage in NPC cells.

Since apoptosis is a critical cellular response to RT,^34^ the apoptosis rate was verified by flow cytometry assay. And we demonstrated an inverse correlation between SALL4 expression and the apoptotic ratio in cells after irradiation (Figure 4C, D). SALL4 silencing markedly increased cell apoptosis in CNE2 and CNE2R cells compared to control groups (Figure 4C, D). In CNE2 groups, the apoptosis rates of the control, shSALL4, RT, and combination of shSALL4 and RT groups were 5.67 ± 0.34, 16.50 ± 1.63, 61.03 ± 3.08, and 85.27 ± 2.13%, respectively (Figure 4C). Similar results were showed in CNE2R groups, the apoptosis rates of the control, shSALL4, RT, and combination groups were 4.77 ± 0.61, 8.47 ± 1.02, 28.00 ± 1.63, and 52.87 ± 6.86%, respectively (Figure 4D). The combination of shSALL4 and RT significantly increased the apoptosis rates. Besides, SALL4 overexpression reduced radiation‐induced apoptosis. The apoptosis rates of the combination of SALL4 overexpression and RT group were significantly lower than that of the RT group (20.70 ± 1.20 vs 61.03 ± 3.08, *P *<* *0.001) in CNE2 groups (Figure 4C).

And the cell cycle was analyzed, too. As shown in Figure 4E, the inhibition of SALL4 increased the G2/M cell populations in CNE2 cells with or without irradiation. The proportions of cells in the G2/M phase within the shSALL4, RT, and combination groups were 25.37 ± 0.34, 28.46 ± 0.38, and 31.39 ± 0.40%, respectively, significantly higher compared with that of the control group (19.91 ± 0.58%). The G2/M proportion of cells with shSALL4 + RT treatment was remarkably higher than that of RT‐alone group (31.39 ± 0.40% vs 28.46 ± 0.38, *P *<* *0.001). Additionally, SALL4 overexpression reduced radiation‐induced G2/M cell populations. The G2/M proportion of cells within the RT+SALL4 overexpression was 21.31 ± 0.27%, significantly lower compared with that of the RT‐alone group (28.46 ± 0.38%) (Figure 4E, *P* < 0.01). In CNE2R cells, no significant differences in G2/M proportion were observed in CNE2R‐shSALL4 cells before irradiation. However, after exposing to RT, silencing SALL4 increased radiation‐induced G2/M arrest in CNE2R cells (Fig. 4F). Thus, inhibition of SALL4 could enhance radiation‐induced DNA damage, cellular apoptosis, and G2/M arrest, reversing the intrinsic and acquired radioresistance of NPC cells.

### 3.5 SALL4 correlates with the expression of proteins related to the ATM/CHK2/P53 pathway

**Figure 5:**
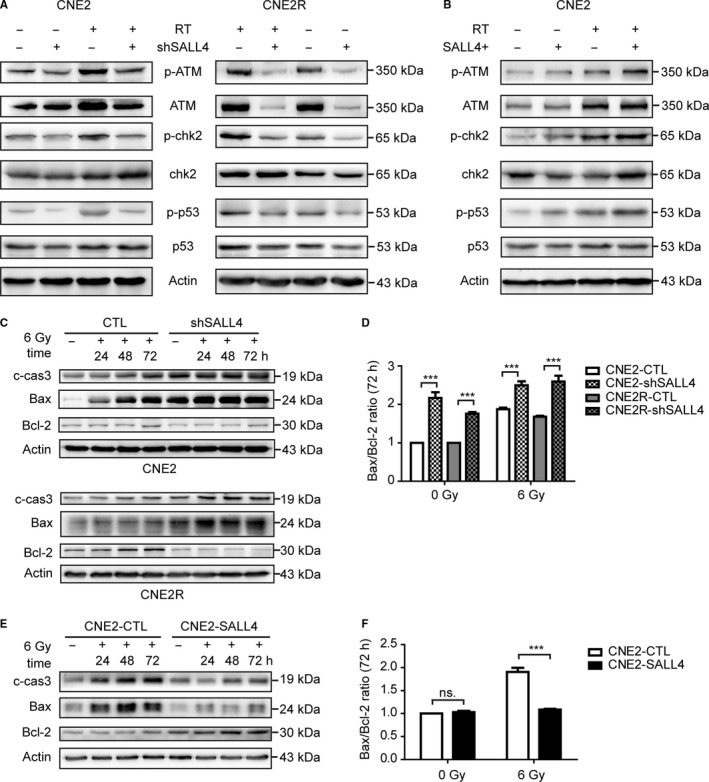
SALL4 correlates with the expression of proteins related to the ATM/CHK2/P53 pathway. Western blot was applied to analyze the expression levels of related proteins. β‐actin was used as a loading control. A, Before and after irradiation, SALL4 silencing reduced the expressions of p‐ATM, p‐Chk2, and p‐p53 in CNE2 and CNE2R cells. B, SALL4 overexpression increased expression of p‐ATM, p‐Chk2, and p‐p53 in CNE2 cells. C, Expressions of pro‐apoptosis proteins (cleaved‐caspase‐3 and Bax) were increased and expressions of anti‐apoptosis protein (Bcl‐2) were decreased in CNE2‐shSALL4 and CNE2R‐shSALL4 cells after irradiation. D, SALL4 silencing increased the Bax/Bcl‐2 ratio. E, Expressions of cleaved‐caspase‐3 and Bax were decreased and expression of Bcl‐2 was increased in CNE2‐SALL4 cells after irradiation. F, SALL4 overexpression reduced Bax/Bcl‐2 ratio in CNE2 cells after radiation ***P <* *0.001. ns: not significant

To explore the signaling pathway by which SALL4 performed its functions, core elements of DDR network (ATM, CHK2, and p53) and their phosphorylated variants were detected by western blot (Figure 5A, B). The expression levels of pro‐apoptotic proteins cleaved‐caspase‐3 and Bax and anti‐apoptotic protein Bcl‐2 were detected as well (Figure 5C, E). Before and after irradiation, SALL4 silencing leads to reduced expression of p‐ATM, p‐Chk2, and p‐p53 in both CNE2 and CNE2R cells (Figure 5A). SALL4 overexpression resulted in increased expression of p‐ATM, p‐Chk2, and p‐p53 in CNE2 cells (Figure 5B). Moreover, expressions of cleaved‐caspase‐3 and Bax were enhanced and increased in a time‐dependent manner in CNE2‐shSALL4 cells and CNE2R‐shSALL4 cells after irradiation (Figure 5C). While the anti‐apoptosis protein Bcl‐2 was downregulated. Accordantly, SALL4 overexpression decreased the expression of pro‐apoptosis protein cleaved‐caspase‐3 and Bax, while Bcl‐2 was upregulated in CNE2‐SALL4 cells (Figure 5E). It is known that apoptosis is governed by the balance of Bax and Bcl‐2,^35^ then the Bax/Bcl‐2 ratio was analyzed (Figure 5D, F). As shown in Figure 5D, SALL4 silencing increased the Bax/Bcl‐2 ratio, no matter NPC cells were irradiated or not. And after exposing to radiation, CNE2‐SALL4 cells showed a decreased Bax/Bcl‐2 ratio (Figure 5F). Together, SALL4 regulated the intrinsic and acquired radioresistance via ATM/Chk2/p53 pathway and its downstream proteins related to apoptosis in NPC.

### 3.6 SALL4 affects the radiosensitivity of NPC in vivo

**Figure 6:**
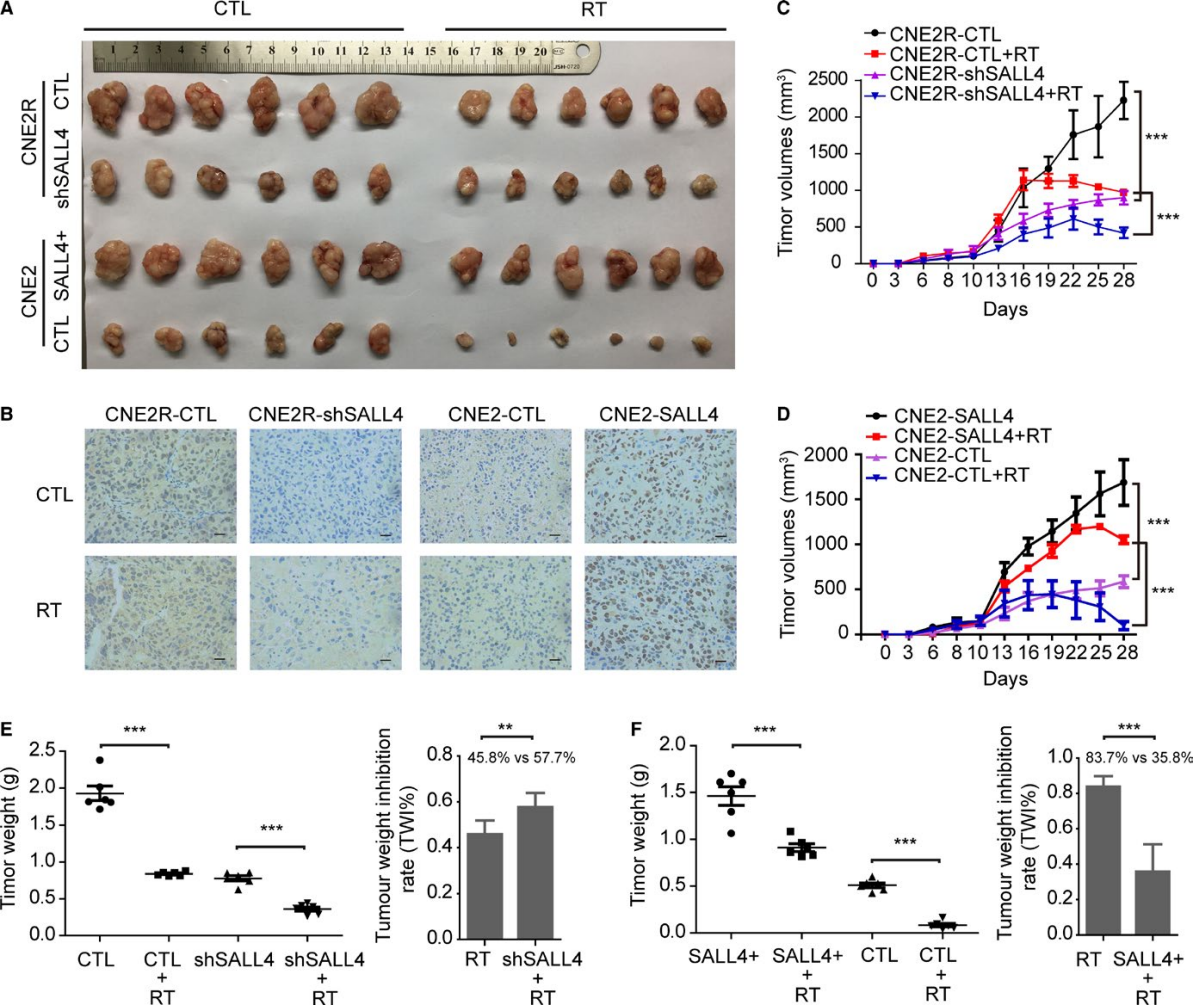
SALL4 affects the tumorigenesis and radiosensitivity of nasopharyngeal carcinoma in vivo. Human NPC xenografts in nude mice model are used. A, Representative tumor xenografts of each group. B, Representative immunohistochemistry staining image for SALL4 expression in tumor tissue. Original magnification × 400. Bar. 20 μm. C, The volumes of tumor in CNE2R‐CTL and CNE2R‐shSALL4 groups treated with or without radiation. D, The volumes of tumor in CNE2‐CTL and CNE2‐SALL4 groups treated with or without radiation. E, The tumor weight (left) and the tumor weight inhibition rate (TWI %) (right) of CNE2R‐CTL and CNE2R‐shSALL4 groups treated with or without radiation. F, The tumor weight (left) and the tumor weight inhibition rate (TWI %) (right) of CNE2‐CTL and CNE2‐SALL4 groups treated with or without radiation. Quantifications of tumor volumes were showed with means ± SD from three independent experiments. ***P <* *0.001, **P <* *0.01

Our in vitro findings indicated that SALL4 overexpression induced radioresistance and SALL4 silencing reversed radioresistance of NPC cells. For further confirmed these observations in vivo, the xenograft experiments in nude mice were conducted. As compared to control groups, the CNE2R‐shSALL4 tumors were much smaller, and CNE2‐SALL4 tumors were larger. (Figure 6A, C and D). Immunohistochemistry of the tumors confirmed lighter staining of SALL4 in CNE2R‐shSALL4 tumors, and darker staining of SALL4 in CNE2‐SALL4 tumors (Figure 6B). After treatments of 8 Gy irradiation, consistent with results in vitro, the growth rate of CNE2R‐shSALL4 tumors was relatively hindered, as compared to the CNE2R tumors (*P *<* *0.001, Figure 6C). And the CNE2‐SALL4 tumor grew obviously faster than the CNE2 tumors after irradiation (*P *<* *0.001, Figure 6D). In addition, irradiation showed a strong inhibitory effect on the tumor weight (*P *<* *0.01, Figure 6E, F). The TWI% of irradiation was relatively higher in CNE2R‐shSALL4 cells than that in CNE2R cells (57.7 ± 5.6% vs 45.8 ± 5.6%, *P *<* *0.01, Figure 6E), besides, the TWI% of irradiation was significantly lower in CNE2‐SALL4 cells than that in CNE2 cells (35.8 ± 14.2% vs 83.7 ± 5.6%, *P *<* *0.001, Figure 6F). Summarily, these outcomes demonstrated that SALL4 induced radioresistance of NPC in vivo.

## 4 DISCUSSION

SALL4 is a crucial factor for the maintenance of self‐renewal and pluripotency of ESCs, decreased gradually during the embryo development and silenced with the maturation of tissues but restored in both hematological diseases and solid tumors.^36^, ^37^ Although aberrant expression of SALL4 has been reported influencing the development and prognosis of various cancers,^38^, ^39^ the role of SALL4 in NPC remains unclear. In this study, we reported that SALL4 expression was strikingly upregulated in NPC tissues compared with noncancerous nasopharyngeal tissues (nasal polyp), and NPC patients in T3‐4 classification often showed higher expression of SALL4. Our results were consistent with previous studies on the expression of SALL4 in other types of cancers.^38^ Additionally, studies also indicated that radiotherapy alone could successfully control less T3‐4 tumors than T1‐2 tumors, meaning T3‐4 tumors might be more resistant to radiotherapy.^40^ Thus, a high expression of SALL4 might compose an adverse prognostic factor for the survival and radiosensitivity of NPC patients. To verify the function of SALL4 in cancer progression, we provided evidence that lower proliferation ability was observed in SALL4 silencing NPC cells. Moreover, SALL4 expression was related to Ki‐67 expression, which is a major biomarker for fast cancer cell proliferation.^41^ These results implied that SALL4 was a pivotal mediator in the progression and prognosis in NPC patients. However, because the samples were collected from 2015, time was not long enough to apply survival analyses, which is a limitation of this study, and the survival analyses are needed to be accomplished in the future.

Aside from the poor prognostic value of SALL4 expression, the impact of SALL4 on radioresistance remains obscure. Our study found that SALL4 silencing caused deteriorated cell survival after RT, whereas, SALL4 overexpression in CNE2 dramatically increased cell survival in vivo and in vitro, suggesting a substantial correlation between SALL4 expression and radioresistance in NPC. We further explored the possible mechanisms that might be involved in SALL4‐induced radioresistance. Previous studies demonstrated that inhibition of SALL4 reduced the chemoresistance through induction of cellular apoptosis in lung and colorectal cancer.^42^, ^43^, ^44^ Besides, ChIP assay found that SALL4 directly bound to the promoter of genes that are critically involved in apoptosis in leukemic NB4 cells.^6^ Also, SALL4 is essential for G1 cell cycle arrest caused by oncoprotein MLL‐AF9 in mixed lineage leukemia rearranged leukemia.^45^ However, there is no relevant study focusing on the role of SALL4 in radiation‐induced DDR. Our study observed a higher level of DNA damage, increased percentages of apoptotic cell, and G2/M cell cycle arrest in SALL4 silencing cells following radiation, and contrary results were observed in SALL4 overexpression cells. Cellular responses to DNA damage, including cell cycle arrest and apoptosis, are important determinants of cancer outcomes following radiation therapy.^46^ Here, we found that inhibition of SALL4 contributed to sensitize NPC cells to radiation, along with enhanced apoptotic cells, DNA damage, and G2/M arrest, indicating that SALL4 regulates the radiosensitivity by impairing radiation‐induced DNA damage and cellular apoptosis. Hence, SALL4 could be a promising therapeutic target for enhancing RT efficiency and prolonging survival in NPC patients.

The present and previous studies confirmed the functional role of SALL4 in DNA damage, apoptosis, and cell cycle arrest, but the signaling pathway needed to be elaborated by which SALL4 regulates the DNA damage response activated by radiation. ATM/CHK2/p53 pathway was a key component among DDR network.^20^ In response to DNA damage, cells activate the sensor kinase ATM that in turn phosphorylates multiple downstream substrates, including Chk2 and p53, resulting in cell cycle checkpoint initiation and/or apoptosis.^47^, ^48^ Since the efficient recruitment and activation of ATM are guaranteed by the interaction of SALL4 with Rad50 to stabilize the Mre11‐Rad50‐Nbs1 complex,^14^ it is reasonable to hypothesize that SALL4 could affect the radiosensitivity via ATM/Chk2/p53 pathway. In both CNE2 and CNE2R cells, with or without radiation exposure, SALL4 silencing vastly downregulated the phosphorylated variants of ATM, Chk2, and p53, and the anti‐apoptosis protein Bcl‐2. Meanwhile, pro‐apoptosis proteins cleaved‐caspase‐3 and Bax were upregulated. Taken together, our findings demonstrate that interference of SALL4 expression could affect the expression of proteins related to the ATM/CHK2/P53 pathway.

In summary, the present study displays the closed correlation among SALL4, radioresistance, and ATM/Chk2/p53 pathway in NPC cells. Our outcomes provide new insights into the mechanism of DNA damage response in radioresistance, which suggest that inhibition of SALL4 have the potential to reverse the intrinsic and acquired radioresistance via inhibiting ATM/Chk2/p53 pathway in NPC patients.

## CONFLICT OF INTEREST

The authors declare that they have no conflict of interest.
